# Nitrogen–potassium balance improves leaf photosynthetic capacity by regulating leaf nitrogen allocation in apple

**DOI:** 10.1093/hr/uhad253

**Published:** 2023-11-27

**Authors:** Xinxiang Xu, Xu Zhang, Wei Ni, Chunling Liu, Hanhan Qin, Yafei Guan, Jingquan Liu, Ziquan Feng, Yue Xing, Ge Tian, Zhanling Zhu, Shunfeng Ge, Yuanmao Jiang

**Affiliations:** College of Horticulture Science and Engineering, Shandong Agricultural University, Tai’an 271018, Shandong, China; Apple Technology Innovation Center of Shandong Province, Tai’an 271018, Shandong, China; Yantai Academy of Agricultural Sciences, Institute of Pomology, Yan’tai 265500, Shandong, China; Yantai Academy of Agricultural Sciences, Institute of Pomology, Yan’tai 265500, Shandong, China; College of Horticulture Science and Engineering, Shandong Agricultural University, Tai’an 271018, Shandong, China; Apple Technology Innovation Center of Shandong Province, Tai’an 271018, Shandong, China; College of Horticulture Science and Engineering, Shandong Agricultural University, Tai’an 271018, Shandong, China; Apple Technology Innovation Center of Shandong Province, Tai’an 271018, Shandong, China; College of Horticulture Science and Engineering, Shandong Agricultural University, Tai’an 271018, Shandong, China; Apple Technology Innovation Center of Shandong Province, Tai’an 271018, Shandong, China; College of Horticulture Science and Engineering, Shandong Agricultural University, Tai’an 271018, Shandong, China; Apple Technology Innovation Center of Shandong Province, Tai’an 271018, Shandong, China; College of Horticulture Science and Engineering, Shandong Agricultural University, Tai’an 271018, Shandong, China; Apple Technology Innovation Center of Shandong Province, Tai’an 271018, Shandong, China; College of Horticulture Science and Engineering, Shandong Agricultural University, Tai’an 271018, Shandong, China; Apple Technology Innovation Center of Shandong Province, Tai’an 271018, Shandong, China; College of Horticulture Science and Engineering, Shandong Agricultural University, Tai’an 271018, Shandong, China; Apple Technology Innovation Center of Shandong Province, Tai’an 271018, Shandong, China; College of Horticulture Science and Engineering, Shandong Agricultural University, Tai’an 271018, Shandong, China; Apple Technology Innovation Center of Shandong Province, Tai’an 271018, Shandong, China; College of Horticulture Science and Engineering, Shandong Agricultural University, Tai’an 271018, Shandong, China; Apple Technology Innovation Center of Shandong Province, Tai’an 271018, Shandong, China; College of Horticulture Science and Engineering, Shandong Agricultural University, Tai’an 271018, Shandong, China; Apple Technology Innovation Center of Shandong Province, Tai’an 271018, Shandong, China; College of Horticulture Science and Engineering, Shandong Agricultural University, Tai’an 271018, Shandong, China; Apple Technology Innovation Center of Shandong Province, Tai’an 271018, Shandong, China

## Abstract

Nitrogen (N) and potassium (K) are two important mineral nutrients in regulating leaf photosynthesis. However, the influence of N and K interaction on photosynthesis is still not fully understood. Using a hydroponics approach, we studied the effects of different N and K conditions on the physiological characteristics, N allocation and photosynthetic capacity of apple rootstock M9T337. The results showed that high N and low K conditions significantly reduced K content in roots and leaves, resulting in N/K imbalance, and allocated more N in leaves to non-photosynthetic N. Low K conditions increased biochemical limitation (*B*_L_), mesophyll limitation (*M*_CL_), and stomatal limitation (*S*_L_). By setting different N supplies, lowering N levels under low K conditions increased the proportion of water-soluble protein N (*N*_w_) and sodium dodecyl sulfate-soluble proteins (*N*_s_) by balancing N/K and increased the proportion of carboxylation N and electron transfer N. This increased the maximum carboxylation rate and mesophyll conductance, which reduced *M*_CL_ and *B*_L_ and alleviated the low K limitation of photosynthesis in apple rootstocks. In general, our results provide new insights into the regulation of photosynthetic capacity by N/K balance, which is conducive to the coordinated supply of N and K nutrients.

## Introduction

Potassium (K) plays significant roles in membrane potential regulation, stress adaption, enzyme activation, and other important physiological processes [1–3]. K also participates in photosynthesis phosphorylation, stomatal closing and opening, and transportation of photosynthetic products, thus regulating quality and crop yield [4, 5]. Therefore, appropriate K supply is an important prerequisite for ensuring normal plant growth and improving crop yield and quality. However, the availability of the natural K in the soils will depend on the intensity of processes like weathering and leaching. The available K level in most soils is gradually decreasing. The lack of K in the soil has become a major limiting factor for sustainable agricultural production [6]. An important reason for soil K deficiency is the high cost of K fertilizer. In recent years, the cost of K fertilizer has increased due to increased input costs, supply chain disruption, and the export restrictions of producers. This will further increase the pressure on small-scale growers in developing countries.

Low K stress has received a great deal of attention as a common abiotic stress that limits crop yield and quality. One of the hazards of low K stress is the severe inhibition of C and N metabolism. Photosynthesis is a process in which plants convert carbon dioxide into carbohydrates, which accounts for 90% of plant biomass. Therefore, maintaining good photosynthesis is conducive to plant growth [7]. As the raw material for photosynthesis, atmospheric CO_2_ needs to diffuse from the leaf surface to the stomata, and then finally reach the carboxylation site of the Rubisco enzyme through the mesophyll cells for photosynthesis. The diffusion process of CO_2_ must overcome the resistance of the leaf surface boundary layer, the resistance of stomata to CO_2_ transport, and the resistance of CO_2_ from the stomatal cavity to the chloroplast Rubisco carboxylation site, in turn. The reduction in photosynthesis due to K deficiency is the result of the combined action of stomatal conductance limitation (*S*_L_), leaf conductance limitation (*M*_CL_), and biochemical limitation (*B*_L_) [8]. In addition, K deficiency inhibits photosynthesis by reducing the number of leaves, decreasing leaf area, decreasing C-metabolizing enzyme activities, and reducing the rate of photosynthetic product assimilation and export, resulting in a severe limitation of C metabolism [9–11]. Recent studies have found that inappropriate N/K would hinder protein synthesis, destroy the stability of mesophyll cells [12], increase the incidence rate and severity of apple canker disease [13], also hinder N assimilation of apples, and reduce photosynthetic nitrogen utilization efficiency [14]. Therefore, maintaining appropriate N/K is of great significance for maintaining plant growth and crop quality.

N is another macronutrient essential for plant growth. It is a key structural component of proteins, amino acids, and chlorophyll, a basic essential element involved in photosynthesis and metabolism, and a major limiting factor for crop yield [3, 15]. N supply levels play significant regulatory roles in C and N metabolism. Furthermore, N supply levels have a direct regulatory effect on the expression of nitrate transporter proteins, with low N (<1 mM) inducing the expression of low-affinity transporter systems and high N inducing the expression of high-affinity transporter systems [16]. N levels also affected N allocation within plants. In maize, Mu *et al*. [17] found that low N increased the proportion of N allocated to electron transport and photosynthetic phosphorylation components. Hou *et al*. [18] reported that high N reduced the proportion of leaf N allocated to the electron transport and carboxylation system components and decreased photosynthetic N utilization efficiency (PNUE) in rice. C assimilation is also influenced by leaf N content, because the enzymatic reactions of C metabolism, the capture of light energy, and electron transport processes require the investment of large amounts of N. Studies have shown that photosynthetic capacity is mainly determined by relative N allocation in leaves. In certain adverse environments, plants usually increase N allocation to the cell wall to improve the plant’s tolerance to adversity [19]. Small changes in N distribution also strongly affect PNUE and photosynthesis.

**Figure 1:**
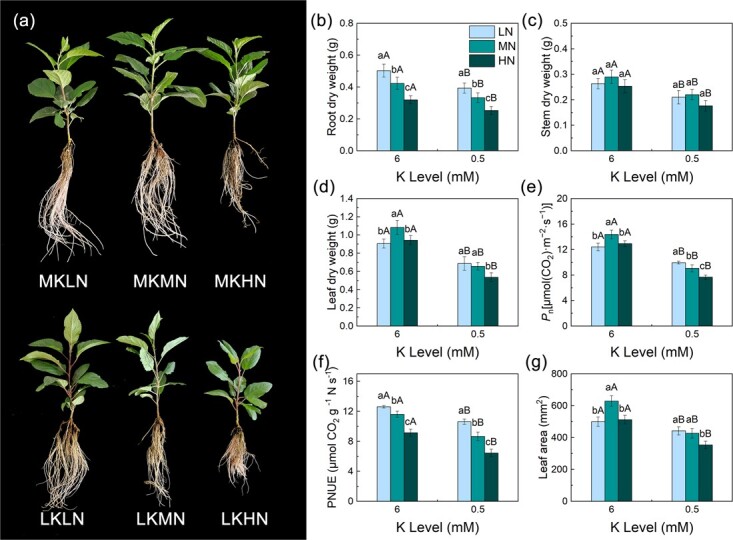
Effects of different N and K levels on M9T337 rootstock growth and photosynthesis. Growth phenotypes of rootstock **(a)**, root dry weight **(b)**, stem dry weight **(c)**, leaf dry weight **(d)**, net photosynthetic rate **(e)**, PNUE **(f)**, and leaf area **(g)**. Data are means ± standard deviation (*n* = 3). Different capital (lower case) letters indicate statistical differences between K (N) levels under the same N (K) level (*P* < 0.05) according to Duncan’s test.

**Figure 2:**
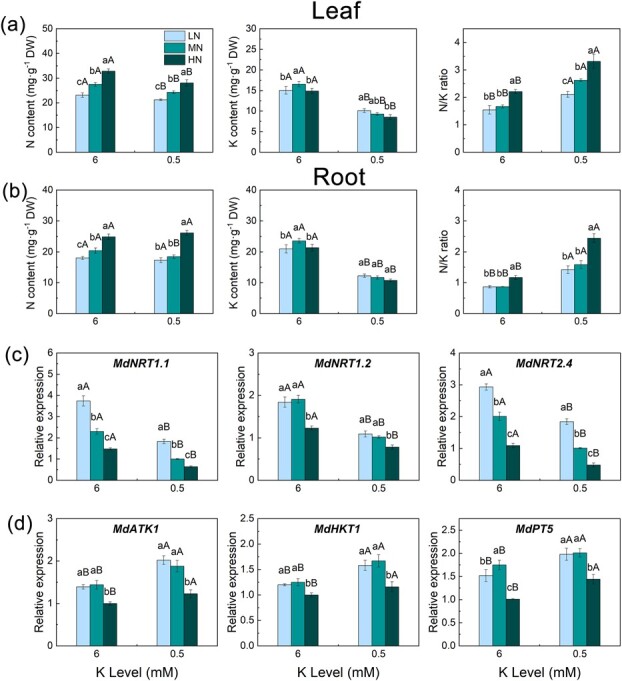
Element content and the relative expression of N and K uptake genes in rootstock roots treated with different N and K levels. N content, K content, and N/K ratio in the leaves **(a)**, N content, K content, and N/K ratio in the roots **(b)**, expression of N uptake genes in roots **(c)**, and expression of K uptake genes in roots **(d)**. Data are means ± standard deviation (*n* = 3). Different capital (lower case) letters indicate statistical differences between K (N) levels under the same N (K) level (*P* < 0.05) according to Duncan’s test.

Recent studies have shown that plant growth balance can be maintained by regulating N supply under stress conditions. Sun *et al*. [20] found that reducing N supply increased the expression of *MdAHA2*, promoted rhizosphere acidification, increased Fe uptake efficiency, and alleviated Fe deficiency in apples. Wen *et al*. [21] found that reducing N supply enhanced the citric acid cycle, increased the content of glucose and sucrose, and thus improved the low light tolerance of tall fescue. Dziedek *et al*. [22] found that N application increased the negative impact of drought on beech. Peng *et al*. [23] found that increasing nitrate supply partially restored the growth retardation and leaf atrophy caused by magnesium deficiency in soybean. These results show that the regulation of N levels under stress conditions is species-specific, and the biological mechanism of N supply in stress tolerance remains controversial. N is one of the elements most closely related to K, but there are few reports on the mechanisms of regulation of low K by N levels in apples.

Apples have a huge planting area and extremely high production in China. However, due to the low price of N fertilizer and its significant effect on yield, fruit farmers tend to overuse N fertilizer in production [24], ignoring the role of K, and the imbalance of N and K nutrition has become an important factor limiting apple production. Mitigating the adverse effects of N and K imbalance on photosynthesis by optimizing N levels may be a feasible option, but the relevant physiological mechanisms have not been specifically analysed. M9T337 rootstock is currently the most common rootstock used in dense dwarf apple production. Therefore, we investigated the effect of different nitrate levels on the growth and C and N metabolism of M9T337 rootstock under different K levels. In this study, we hypothesized that: N/K imbalance would alter leaf N allocation; optimization of N allocation in leaves would improve photosynthesis. Our results provide new clues to the rational application between N and K in apples and other species.

## Result

### Plant growth and photosynthesis

Low K (LK) treatments significantly inhibited the root and aboveground growth of plant (Fig. 1). Compared with medium K (MK) treatments, root dry weight was reduced by 21.85, 21.26, and 20.83% and leaf dry weight by 19.85, 35.14, and 40.99% under the LKLN, LKMN, and LKHN treatments, respectively. Under the MK treatments, the biomass of M9T337 rootstock increased and then decreased as the level of nitrate supply increased, and the plant grew best under the MN treatment. Under the LK treatments, apple rootstock leaf dry weight and root dry weight were the highest under the LN treatment.

K deficiency significantly reduced the leaf area, *P*_n_, and PNUE. Under MK treatments, the leaf area and *P*_n_ were highest under MN treatment, while there was no significant difference between the HN and LN treatments in the leaf area and *P*_n_. Under the LK treatments, the leaf area and *P*_n_ under the LN treatment were the highest, and the leaf area is increased by 3.51 and 24.90%, respectively, compared with the MN and HN treatments, and the *P*_n_ increased by 9.78 and 29.73%, respectively. The results of PNUE showed that with the increase in the N level and the decrease in the K level, PNUE decreased gradually.

### N and K uptake

As shown in Fig. 2a, the LK treatments significantly reduced the N and K content of rootstock leaves but increased the N/K ratio. Under the MK treatment, the highest K content in the roots and leaves was in the MN treatment, and the highest N content was in the HN treatment. However, under the LK treatment, the highest K content and lowest N content were found in rootstock leaves under the LN treatment. The N/K ratio decreased with increasing N levels, and the lowest leaf N/K ratio was found under the HNLK treatment. The patterns of N and K content and N/K ratio in the roots were similar to those in the leaves (Fig. 2b).

We determined the expression of *MdNRT1*.*1*, *MdNRT1*.*2*, and *MdNRT2*.*4* in the roots (Fig. 2c). The results showed that the LN treatments inhibited the transcription levels of nitrate transporters (*NRTs*) and increased the transcription levels of *MdAKT1*, *MdHKT1*, and *MdPT5*. Compared with the MN and HN treatments, the LN treatments increased the expression levels of *MdNRT1.1* and *MdNRT1.2*. The HN treatments decreased the expression levels of *MdAKT1*, *MdHKT1*, *MdPT5*, *MdNRT1.1*, *MdNRT1.2*, and *MdNRT2.4*.

We used ^15^N to mark the N distribution in the rootstocks. The LK treatments significantly reduced the ^15^N distribution rate of leaves and increased the ^15^N distribution rate of roots (Fig. S1a, see online supplementary material). Under the LK treatments, compared with the LKMN and LKHN treatments, the ^15^N distribution rate of leaves in the LKLN treatment increased by 9.28 and 33.95%, respectively, while that of roots decreased by 4.30 and 12.29%, respectively. We also determined the expression of *MdNRT1.5* in roots (Fig. S1, see online supplementary material), which is related to the transport of NO_3_^−^ to the aboveground parts. The LKLN treatment showed higher *MdNRT1*.*5* expression in the roots than the LKHN and LKMN treatments (Fig. S1b, see online supplementary material).

### N allocation by form in leaves

**Figure 3:**
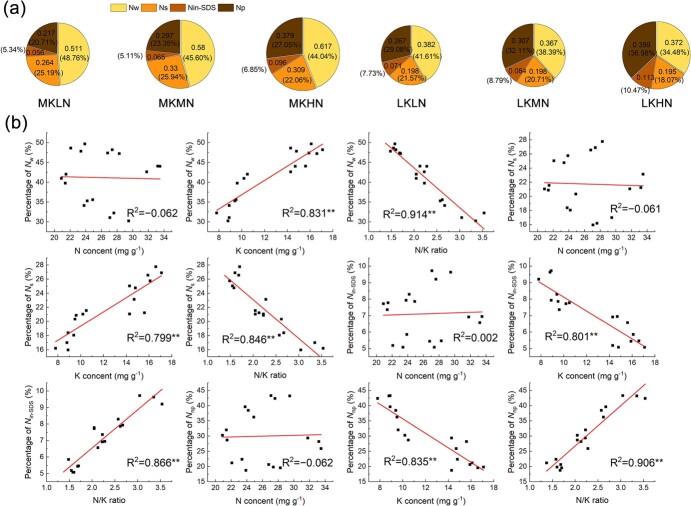
Effects of different N and K levels on N distribution of M9T337 rootstock leaves. N allocation by morphology **(a)** and relationship between N allocation and N content, K content, and N/K in leaves **(b)** of M9T337 rootstock treated with different N and K levels. Data out of the bracket represents the absolute N content (g m^−2^). Data in the bracket represents the relative content (%). The size of pie chart indicates N content.

We analysed the allocation of N forms in leaves under different N and K treatments (Fig. 3b). The LK treatments significantly reduced the content and proportion of water-soluble proteins (*N*_w_) and SDS-soluble proteins (*N*_s_), but they increased the content and composition of SDS-insoluble proteins (*N*_in-SDS_) and non-protein N (*N*_p_). Under the LK treatments, the HN treatment further reduced the content and composition of *N*_w_ and *N*_s_, and increased the content and composition of *N*_in-SDS_ and *N*_p_. Compared with the LKMN and LKHN treatments, the LKLN treatment had the highest content and composition of *N*_w_ and *N*_s_. We conducted a correlation analysis between the allocation proportion of different N forms and N content, K content, and the N/K ratio in the leaves (Fig. 3c). The allocation proportion of different N forms had no significant correlation with the N content in the leaves. The allocation proportion of *N*_w_ and *N*_s_ was positively correlated with K content and negatively correlated with the N/K ratio, while the allocation proportion of *N*_in-SDS_ and *N*_np_ was significantly positively correlated with K content and the N/K ratio.

### Photosynthetic N allocation in leaves

**Figure 4:**
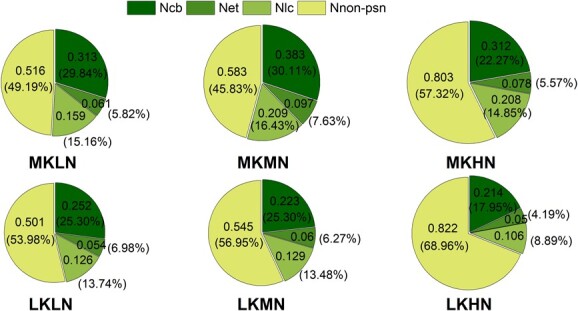
Photosynthetic N allocation of M9T337 rootstock leaves treated with different N and K levels. Data out of the bracket represents the absolute N content (g m^−2^). Data in the bracket represents the relative content (%). The size of pie chart indicates N content.

The allocation of photosynthetic N and non-photosynthetic N in leaves was further analysed (Fig. 4). The LK treatments significantly reduced the content and composition of electron transfer N (*N*_et_), light capture N (*N*_lc_), and carboxylation N (*N*_cb_) and increased the content and composition of non-photosynthetic N (*N*_non-psn_). Under the LK treatments, the photosynthetic N allocation in leaves under the LN treatment was the highest, reaching 46.34%, followed by the LKMN treatment at 41.18%, and the LKHN treatment was the lowest, at only 31.05%. Compared with the LKLN and LNMN treatments, the absolute content of *N*_cb_ under the LKHN treatment decreased by 15.23 and 11.82%, respectively, and the absolute content of *N*_lc_ decreased by 16.16 and 17.93%, respectively, while the absolute content of *N*_non-psn_ increased by 63.92 and 33.33%, respectively. These results showed that LN conditions optimized N allocation in plant organs and leaves under LK conditions.

### Correlation analysis

**Figure 5:**
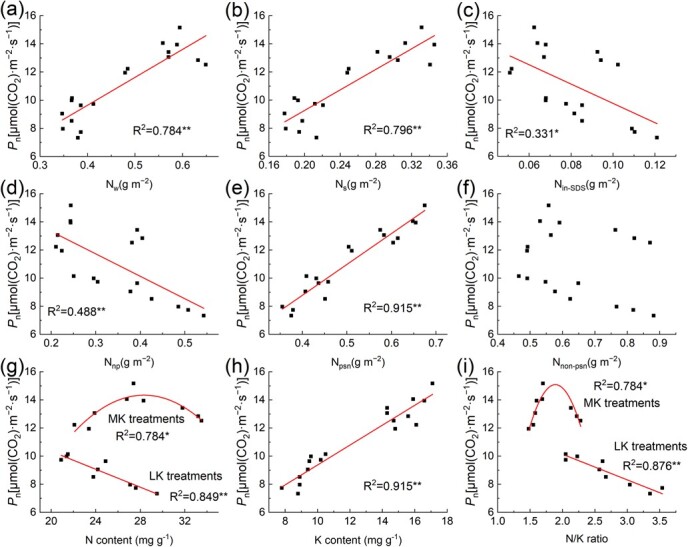
Relationship between the net photosynthetic rate (*P*_n_) and absolute and relative content of the different N forms, N and K content, and N/K.

We analysed the relationship between the absolute and relative content of different N forms and *P*_n_, and the results showed that *P*_n_ was positively correlated with the absolute and relative content of *N*_w_, *N*_s_, and *N*_psn_ (Fig. 5), and negatively correlated with the relative content of *N*_in-SDS_, *N*_np_, and *N*_non-psn_, as well as the absolute content of *N*_in-SDS_ and *N*_np_ (Fig. S2, see online supplementary material). The correlation between the relative content of different N and *P*_n_ was higher than the absolute content of different N forms. We also analysed the relationship between N and K content, N/K and *P*_n_ in leaves. The results showed that *P*_n_ was positively correlated with K content, negatively correlated with N content and N/K under LK treatments. Under MK treatments, with the increase of N content and N/K, the *P*_n_ first increased and then decreased.

### Leaf anatomical and structural traits

**Figure 6:**
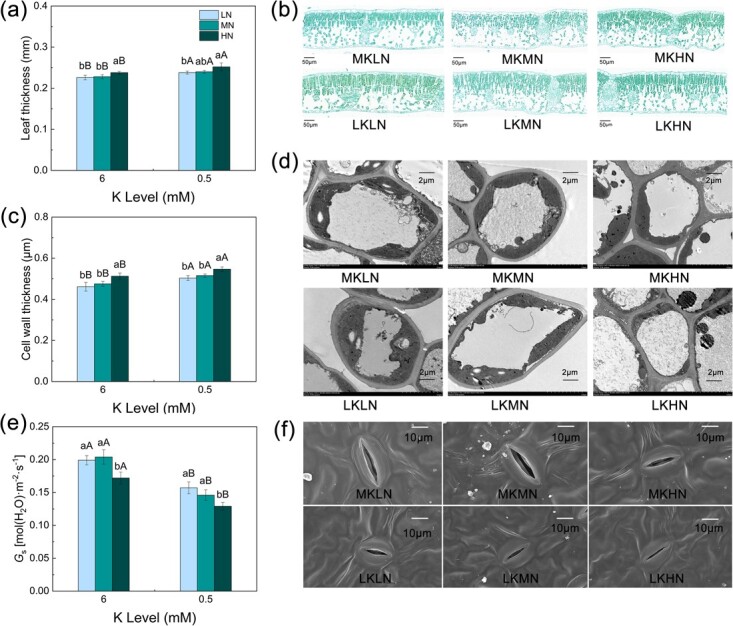
Effects of different N and K levels on leaf structure parameters of M9T337 rootstock. Leaf thickness (**a** and **b**), cell wall thickness (**c** and **d**), and stomatal conductance (**e** and **f**). Data are means ± standard deviation (*n* = 6). Different capital (lower case) letters indicate statistical differences between K (N) levels under the same N (K) level (*P* < 0.05) according to Duncan’s test.

The cell wall thickness and stomatal conductance were significantly influenced by different N and K treatments, but there was no significant difference in leaf thickness (Fig. 6). Under the MK and LK treatments, the cell wall thickness of the HN treatment was significantly higher than those of the LN and MN treatments. Compared with the MKLN and MKMN treatments, the thickness of the cell wall under the MKHN treatment increased by 5.80 and 4.79%, respectively. There was no significant difference in cell wall thickness between the MN and LN treatments. We measured stomatal conductance. Under the MK treatments, the *g*_s_ of the MN treatment was the largest. Compared with the LKMN and LKHN treatments, the *g*_s_ of leaves under the LKLN treatment increased by 16.22 and 29.43%, respectively. N and K also affected the cell arrangement. Fig. 6b shows that the palisade tissue was more closely arranged under the LK and HN treatment.

### Photosynthetic limitation

Under the MK treatments, *g*_sc_, *g*_m_, and *V*_max_ were highest under the MN treatment, and *g*_m_ and *V*_max_ were not significantly different between the LN and HN treatments. Leaf *g*_sc_, *g*_m_, and *V*_max_ increased by 16.22, 9.62, and 4.02%, respectively, under the LKLN treatment compared to the LKMN treatment and by 29.43, 23.32, and 17.96%, respectively, compared to the LKHN treatment. Thus, *S*_L_, *M*_CL_ and *B*_L_ were minimal under the LKLN treatment.

**Figure 7:**
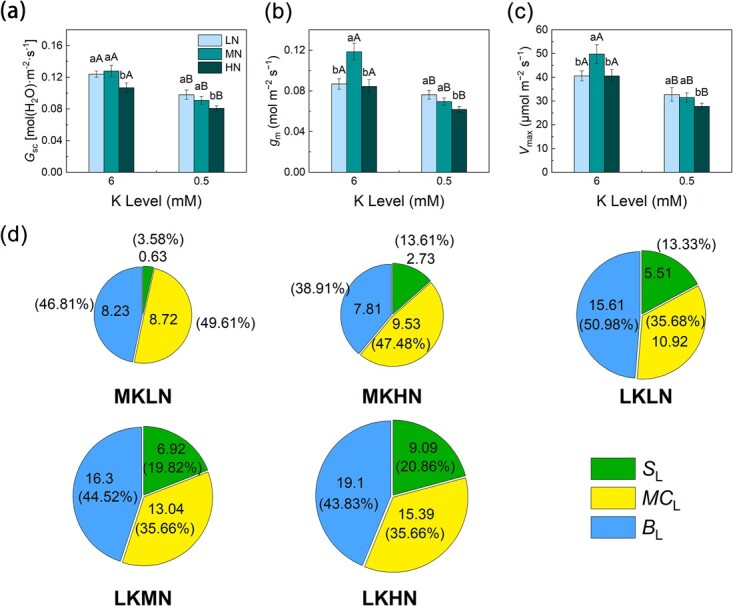
Photosynthesis limitations of M9T337 rootstock treated with different N and K levels. Stomatal conductance to CO_2_  **(a)**, mesophyll conductance **(b)**, the maximum carboxylation rate of Rubisco **(c)**, photosynthesis limitations and components **(d)**. Data out of the bracket represents the measured values. Data in the bracket represents the relative values. Data are means ± standard deviation (*n* = 3). Different capital (lower case) letters indicate statistical differences between K (N) levels under the same N level (*P* < 0.05) according to Duncan’s test. The size of pie chart indicates photosynthetic limitation.

Under the LN treatments, *B*_L_ was the highest limitation of photosynthetic decline in leaves, followed by *M*_CL_, and *S*_L_ was the lowest (Fig. 7). Under the MK treatments, *M*_CL_ was the highest limitation of photosynthetic decline in the leaves.

## Discussion

N and K are essential nutrients for plants and are crucial for their growth and development. K deficiency limits plant growth, which may be related to an imbalance of N and K nutrients [25]. The interactions between N and K are complex and occur at multiple levels [26]. Previous studies have generally concluded that K^+^ and NO_3_^−^ are mutually reinforcing relationships because the charge balance improves [27]. However, the K content in the leaves and the K^+^ influx rate in the roots decreased under the HN treatments, indicating that high nitrate inhibited the K uptake. The same results were reported by Li *et al*. [25], who found that increased N fertilization exacerbated K deficiency, but they attributed this to a mutual dilution effect between N and K. However, in our experiments, the HN treatments did not show an increase in biomass. The HN treatment downregulated the expression of *MdATK1* and *MdPT5*, which explains the inhibition of K uptake by high N. The expression of *MdATK1*, *MdHKT1*, and *MdPT5* was significantly increased under the LK treatments, which is a stress response due to plant K deficiency [28]. Plants actively absorb NO_3_^−^ through NRTs in roots and then synthesize amino acids and proteins under the action of a series of N metabolism enzymes, such as NR and GS [29]. We found that the LK treatments reduced the expression levels of *MdNRT1*.*1* and *MdNRT2*.*4* in roots, thereby inhibiting nitrate uptake. These results are similar to previous findings. The inhibition of N uptake by the LK treatments was exacerbated by high nitrate levels. Compared with the LKLN and LKHN treatments, the NRT expression in roots was reduced significantly under the LKHN treatment, which is consistent with the results of Sun *et al*. [30] on apples. Although low K inhibited N uptake, the N/K ratio in the roots and leaves remained significantly elevated.

N and K levels also affect the N distribution in plants and their leaves. Increasing the aboveground N distribution of the plant and the proportion of photosynthetic N in the leaves can increase PNUE, which has been confirmed in rice and oilseed rape [18, 31]. We labeled N allocation in plants with ^15^N and found that the LK treatments reduced the percentage of aboveground N allocation, which may be related to the fact that low K reduced the expression of *MdNRT1*.*5*, a key transporter protein involved in N loading in the xylem [32]. Under low K conditions, high N treatment further inhibited *MdNRT1*.*5* expression, leading to a further reduction in ^15^N distribution in the leaves, similar to the results found by Wu *et al*. [33]. Leaf N can be divided into *N*_w_, *N*_s_, *N*_in-SDS_, and *N*_p_ [34]. N_in-SDS_ consists mainly of cell wall protein N and nuclear protein N. Plants react to a stressful environment by increasing the amount of cell wall protein N, which has been confirmed in rice [35]. We found that low K conditions reduced the partitioning of *N*_w_ and *N*_s_ and increased the partitioning of *N*_in-SDS_ and *N*_np_, which is similar to the results of Hou *et al*. [18] in rice. We analysed the correlation between N content, K content, and the N/K ratio and the allocation proportions of *N*_w_, *N*_s_, *N*_in-SDS_, and *N*_p_. The correlation coefficient between the N/K ratio and the distribution proportion of different forms of N was the highest, and the N/K ratio was significantly positively correlated with the distribution proportion of *N*_in-SDS_ and *N*_p_. Therefore, under low K conditions, high N further increased the proportion of *N*_in-SDS_ and *N*_np_, which may be related to the increase in the N/K ratio due to high N. The more N allocated to *N*_in-SDS_ and *N*_np_ fractions, the smaller the proportion allocated to *N*_w_ and *N*_s_. Photosynthetic N mainly includes *N*_cb_, *N*_lc_, and *N*_et_, of which *N*_cb_ belongs to *N*_w_, while *N*_lc_ and N_et_ belong to *N*_s_. We further analysed the distribution of photosynthetic N in leaves, with the same results regarding the N form; low K conditions reduced the absolute and relative content of *N*_cb_ and *N*_lc_, leading to a reduction in the proportion of *N*_psn_. We also analysed the relationship between the absolute and relative content of N and *P*_n_. The relationship between the relative content of N and *P*_n_ was closer than the absolute content. Therefore, different N and K treatments regulated photosynthesis by adjusting the N allocation.

As the main osmoregulatory substance of guard cells, the abundance of K^+^ affects the function of stomata; thus, low K conditions usually lead to stomatal closure [36, 37]. We confirmed this result by observing the stomatal structure using a scanning electron microscope. High N further reduced *g*_s_, which may be related to the inhibition of K uptake under high N conditions. In addition, the *g*_m_ from the sub-stomatal cavities to the sites of carboxylation inside the chloroplasts is one of the main factors affecting photosynthesis [36]. Previous studies have shown that in stressful environments, plants tend to have smaller leaves and greater leaf pulp cell density and leaf thickness to resist adversity and prolong leaf life [38]. We also observed that low K conditions reduced the leaf area. The results of paraffin sections also showed that the arrangement of palisade tissues was more compact under K deficiency conditions, which is detrimental to the capture of light energy by the leaves and CO_2_ conduction in the leaves. Hou *et al*. [18] reported that high N treatment led to more leaf N being allocated to non-photosynthetic N. Our results found that the HN treatment aggravated the N/K imbalance, causing plants to allocate more N to *N*_in-SDS_, which are mainly cell wall proteins and nucleoproteins. Therefore, the increase in *N*_in-SDS_ content may indicate an increase in cell wall thickness. We measured the cell wall thickness and found that the LK and HN treatments increased the cell wall thickness. This is similar to the findings of Xie *et al*. [39] on rice. They also found that under the condition of severe K deficiency, the cell wall thickness increased significantly with an increase in N application, thereby reducing the relative content of *N*_psn_. This phenomena can potentially be explained as nutrient stress promotes more N into cell wall structure to reduce the damage to plants from nutrient imbalance [40]. The thickening of the cell wall further limited the diffusion of CO_2_ into the chloroplast, as leaves with strong cell wall structures tended to exhibit a lower g_m_ [7, 41]. Hu *et al*. [12] found that suitable N and K nutrients could coordinate CO_2_ diffusion and carboxylation in rice, thereby enhancing photosynthetic capacity. We found that under LK conditions, the LN treatment balanced N/K by reducing N content and the proportion of *N*_in-SDS_ and *N*_np_, and more N was distributed to *N*_psn_, such as N_cb_. Generally, the higher N allocation to Rubisco, the higher *V*_max_ and subsequently higher *P*_n_ [42]. Our results also showed that nitrogen and potassium balance reduces cell wall thickness and increases *V*_max_, ultimately leading to significant reductions in *M*_CL_ and *B*_L_.

**Figure 8:**
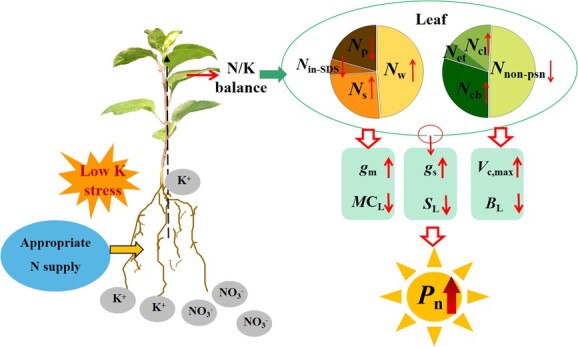
A hypothetical model for regulating photosynthesis of apple rootstock leaves by N/K balance.

In conclusion, the regulation of N and K balance on photosynthesis is complex. The LK treatments leading to an imbalance of N/K, which led to an increase in leaf *N*_in-SDS_ and *N*_np_ allocation and a decrease in *N*_psn_ allocation. The change in leaf N distribution led to the decrease in *g*_m_ and *V*_max_, leading to increased photosynthetic limitation. Under low K conditions, by adjusting the N supply level to coordinate N/K, the relative content of *N*_in-SDS_ was reduced, and plants allocated more N to *N*_s_ and N_psn_, thus increasing PNUE and *V*_max_ and alleviating the limitation of photosynthesis by low K. In conclusion, maintaining the proper ratio of N and K concentrations in leaves by optimizing the N/K ratio could coordinate the distribution of N, reduce photosynthetic restrictions, and improve leaf photosynthetic capacity, thus promoting apple rootstock growth (Fig. 8). Our findings are expected to provide new insights into the regulation of photosynthetic capacity by N/K balance.

## Materials and methods

### Growth conditions and treatments

Apple seedlings (M9T337 dwarf rootstock, *n* = 240) were used in the current study. M9T337 rootstocks were planted in a growth chamber under natural light and temperature conditions. We used a hydroponic system designed as described by Xu *et al*. [43], there were 40 rootstocks in each treatment, and one rootstock was used as a biological repeat. Briefly, consistently grown rootstocks (approximately 10 cm high) were selected and transferred to black plastic containers containing 6 L of 1/2 Hoagland’s nutrient solution [44].

In the pre-experiment, two K supply levels (0.5 and 6 mM) and four N supply levels (0.1, 1, 10, and 20 mM) were set. Apple rootstocks under the 0.1 mM N level showed highly significant N deficiency symptoms, and the growth of aboveground parts was significantly inhibited regardless of the K supply level, while the rootstocks grew best at 1 mM N supply under low K conditions (Fig. S3, see online supplementary material). Thus, we selected three N levels of 1, 10, and 20 mM for treatment in the formal experiment.

The formal trial began after 10 days of growth. Ca(NO_3_)_2_ was used as the only N source and K_2_SO_4_ as the only K source. Two K supply levels (6 and 0.5 mM) and three N supply levels (1, 10, and 20 mM) were used in this study. Six treatments were included: medium K low L (6 mM K^+^ + 1 mM NO_3_^−^, MKLN), medium K medium N (6 mM K^+^ + 10 mM NO_3_^−^, MKMN), medium K high N (6 mM K^+^ + 20 mM NO_3_^−^, MKHN), low K low L (0.5 mM K^+^ + 1 mM NO_3_^−^, LKLN), low K medium N (0.5 mM K^+^ + 10 mM NO_3_^−^, LKMN), and low K high N (0.5 mM K^+^ + 20 mM NO_3_^−^, LKHN). The other compositions of nutrient solution are equal between groups: 0.1 mM EDTA-Fe, 0.76 μM ZnSO_4_·7H_2_O, 37 μM H_3_BO_4_, 0.3 μM CuSO_4_·5H_2_O, 9 μM MnCl_2_·4H_2_O, 1 mM NaH_2_PO_4_, and 2 mM MgSO_4_. CaCl_2_ was supplemented in the LN and MN treatments at the same Ca^2+^ level as in the HN treatment. We adjusted the nutrient solution PH to 6.0 ± 0.1 and replaced the solution every 3 days. An air pump was used to maintain the oxygen content of the nutrient solution (12 h per day). The samples were taken to determine the various indices after 28 days of treatment.

### ^15^N isotope analysis

Ten rootstocks were selected for ^15^N labeling in each treatment. Ca(NO_3_)_2_ was replaced with Ca(^15^NO_3_)_2_ (with an abundance of 10.14%), and other nutrient contents and management were the same as those described above. The rootstocks were destructively sampled and divided into leaves, stems, and roots. After drying at 80°C to constant weight, they were ground and filtered with a mesh screen (0.25 mm). The abundance of ^15^N was analysed using a MAT-251-Stable Isotope Ratio Mass Spectrometer. The formula was calculated following the method of Xu *et al*. [43].

### Analysis of growth parameter and mineral element

After 28 days of treatment, rootstocks were collected to analyse dry weight and root morphology. The leaves, roots, and stems of rootstocks were dried to constant weight at 80°C, and each organ was weighed with a 1/1000 electronic balance. The root morphology was analyzed by WinRhizo software (WinRHIZO version2012b, Regent Instruments Canada, Montreal, QC, Canada).

The dried samples were ground into powder and digested with H_2_SO_4_–H_2_O_2_. The N content was determined using the Kjeldahl apparatus (JK9870). The K content was determined using a flame photometer (M-410; Cole-Parmer, Chicago, IL, USA).

### Determination of leaf characteristics and quantitative limitation analyses of *P*_n_

Chlorophyll was extracted from the leaves and measured according to the method of Porra *et al*. [45]. The leaf area meter (Yaxin-1241, Beijing, China) was used to measure the leaf area.

After 28 days of treatment, gas exchange and *P*_n_ − *C*_i_ curves of the fourth main-stem leaf were determined using a portable photosynthesis system (LI-6400, LI-COR, Lincoln, Nebraska, USA) between 9:00 and 11:30 a.m. The maximum carboxylation efficiency (*V*_max_) and maximum electron transfer rate (*J*_max_) were calculated according to Long and Bernacchi [46]. Mesophyll conductance (*g*_m_) was calculated following Harley *et al*. [47]. *G*_sc_ is stomatal conductance to CO_2_ (*g*_s_/1.6).

The limitations to *P*_n_ mainly consists of the stomatal limitations (*S*_L_), mesophyll conductance limitations (*M*_CL_), and biochemical limitations (*B*_L_) according to the analyses presented by Grassi and Magnai [48]. The formula is calculated following Lu *et al*. [49].

### N allocation by form and function

A punch (10 mm diameter) was used to collect leaf disks (1 cm^2^ area) from the leaves, avoiding the main vein, with 20 pieces for each treatment. The leaf disks were used to determine the different forms of N (Fig. S4, see online supplementary material). Different forms of N (water-soluble protein, *N*_w_; SDS-soluble proteins, *N*_s_; SDS-insoluble proteins, *N*_in-SDS_) in leaves were measured according to Liu *et al*. [50]. The three N components were dried and digested with H_2_SO_4_–H_2_O_2_ [51]. The N content in the digestion solution was determined by Kjeldahl apparatus (JK9870).

According to the results of Niinemets and Tenhunen [52], leaf N consisting of photosynthetic N (*N*_psn_) was divided into three major parts: proteins for carboxylation in the Calvin cycle (*N*_cb_); light-harvesting components (*N*_lc_); and electron transport components (*N*_et_). The calculation formula of the N allocation proportion was as follows:

(1) \begin{equation*} {N}_{psn}={N}_{cb}+{N}_{lc}+{N}_{et};{N}_{non- psn}\left( non- photosynthetic\ N\right)=1-{N}_{psn} \end{equation*}

(2) \begin{align*} \notag&{N}_{cb}=\frac{Vmax}{6.25\times Vcr\times Na}\\ &{V}_{max}\ \mathrm{is}\ \mathrm{the}\ \mathrm{maximum}\ \mathrm{carboxylation}\ \mathrm{efficiency};{V}_{cr}=20.78\ \mathrm{\mu} \mathrm{mol}\notag\\ & C{O}_2{g}^{-1}{\mathrm{Rubiscos}}^{-1};{N}_a\ \mathrm{is}\ \mathrm{the}\ \mathrm{leaf}\ N\ \mathrm{content}\ \mathrm{per}\ \mathrm{unit}\ \mathrm{area}. \end{align*}

(3) \begin{align*} \notag&{N}_{et}=\frac{Jmax}{8.06\times Jmc\times Na}\\ &{J}_{max}\ \mathrm{is}\ \mathrm{maximum}\ \mathrm{electron}\ \mathrm{transfer}\ \mathrm{rate};{J}_{mc}=155.65\kern0.50em \mathrm{\mu} \mathrm{mol}\notag\\ & {\mathrm{e}}^{-}\mathrm{\mu} \mathrm{mol}\ \mathrm{Cyt}\ \mathrm{f}\ {\mathrm{s}}^{-1} \end{align*}

(4) \begin{align*} \notag &{N}_{1c}\frac{Cc}{Cb\times Na}\\ &{C}_c\ \mathrm{is}\ \mathrm{chlorophyll}\ \mathrm{content};{C}_b=2.15\kern0.50em \mathrm{mmol}\ {\mathrm{g}}^{-1}\ N \end{align*}

(5) \begin{align*} &\notag\mathrm{PNUE}=\frac{Pn}{Na}\\ &{P}_n\ \mathrm{is}\ \mathrm{maximum}\ \mathrm{net}\ \mathrm{photosynthetic}\ \mathrm{rate}. \end{align*}

### Leaf structure analysis

The structure of the leaves and the thickness of the leaves were studied using tissue paraffin sectioning. The tissue paraffin sectioning and scanning electron microscopy (SEM) slides of the leaves were prepared according to Xu *et al*. [44]. The transmission electron microscope (TEM) slides of the leaves were prepared according to Xie *et al*. [39]. The leaf thickness and cell wall thickness were analysed with Image-PRO plus 6.0 software (Media Cybernetics, Silver Spring, MD, USA). The measurements were repeated six times for each treatment.

### Gene expression analysis by qRT-PCR

Total RNA was extracted from root samples using an RNAprep Pure Plant Kit (Tiangen, Beijing, China). The reaction system contained 2 μL of primers (1 μL of upstream and 1 μL of downstream primers), 1 μL of cDNA, 10 μL of Green qPCR SuperMix, and 7 μL of ddH_2_O. Data were calculated using the 2^–ΔΔCT^ method [53]. The primers used in the assays are listed in Table S1 (see online supplementary material).

### Data analysis

All statistical analyses were analysed using SPSS (Statistics software, version 17.0, IBM, USA). The post hoc test (Duncan's) were used to test for statistical significance. Differences were deemed significant at *P* ≤ 0.05.

## Acknowledgements

This work was supported bythe National Key R & D Program of China (2023YFD2301000), the Special Fund for the Natural Science Foundation of Shandong Province (ZR2021MC093), the earmarked fund for CARS (CARS-27), the Taishan Scholar Assistance Program from Shandong Provincial Government (TSPD20181206), and Xinlianxin Innovation Center for Efficient Use of Nitrogen Fertilizer (2020-apple).

## Author contributions

S.G., Y.J., and X.X. conceived and designed the experiments. X.X., W.N., Y.G., C.L., Y.X., X.Z., J.L., Z.F., and G.T. performed all experiments. X.X, X.Z., and S.G. analysed the data and wrote the manuscript.

## Data availability

The authors confirm that all experimental data are available and accessible via the main text and/or the supplemental data.

## Conflict of interest statement

The authors declare they do not have any conflict of interest.

## Supplementary data

Supplementary data is available at *Horticulture Research* online.

## Supplementary Material

Material_uhad253
