# Supplementary material for: Nitrogen–potassium balance improves leaf photosynthetic capacity by regulating leaf nitrogen allocation in apple
Source: Hortic Res. 2023 Nov 27;11(1):uhad253. doi: 10.1093/hr/uhad253 (PMC10939330; doi:10.1093/hr/uhad253)
Supplement: Material_uhad253 [file material_uhad253.docx]

**Table S1** | Primer sequences for qRT-PCR.

| Gene  name | Forward sequence of the primers  (5′→3′) | Reverse sequence of the primers  (5′→3′) |
| --- | --- | --- |
| *MdNRT1.1* | CTCGGCCTCATTGTGTTCTT | TCCAACGGCAGTTCCATATTC |
| *MdNRT1.2* | TTAATTGCTGCCACACTTCATAG | CACGATGTTTGGTTCTGATACTTC |
| *MdNRT1.5* | CGGAGGATACCAACCAAACA | GTTCATGGCCAGGTAGAAGTAG |
| *MdNRT2.4* | CGAAGCAAGACATCGGAAATG | TAGCGTGGCCCTATCAAATC |
| *MdHKT1* | GATGTTATTGGGTGGGGAGG | ATTAGAGTCTGGGGGATGGTGT |
| *MdAKT1* | AGGACATGGTTTGGGCTTATC | GGTGGTTGTGAGCGTGATTA |
| *MdPT5* | TCATCGTGCTATGGGCTAATG | CCCTGTCTTCTGGTTGGTTATT |
| *MdActin* | CATGGTTGGTATGGGTCAGAAG | GTCATCCCAGTTGCTCACTATG |


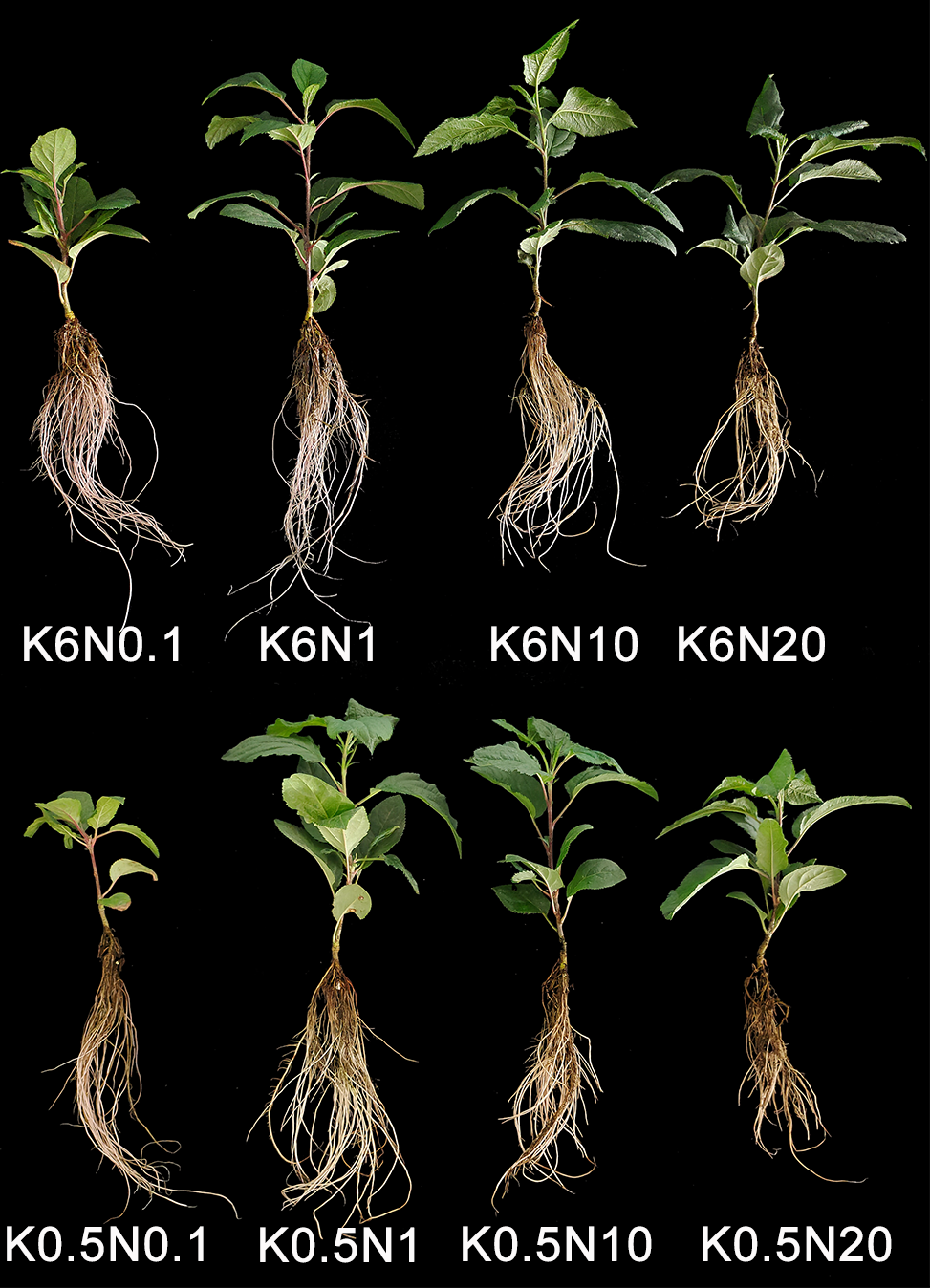


**Figure S1** The growth phenotype of apple rootstock was treated with different nitrogen and potassium in the pre-experiment.


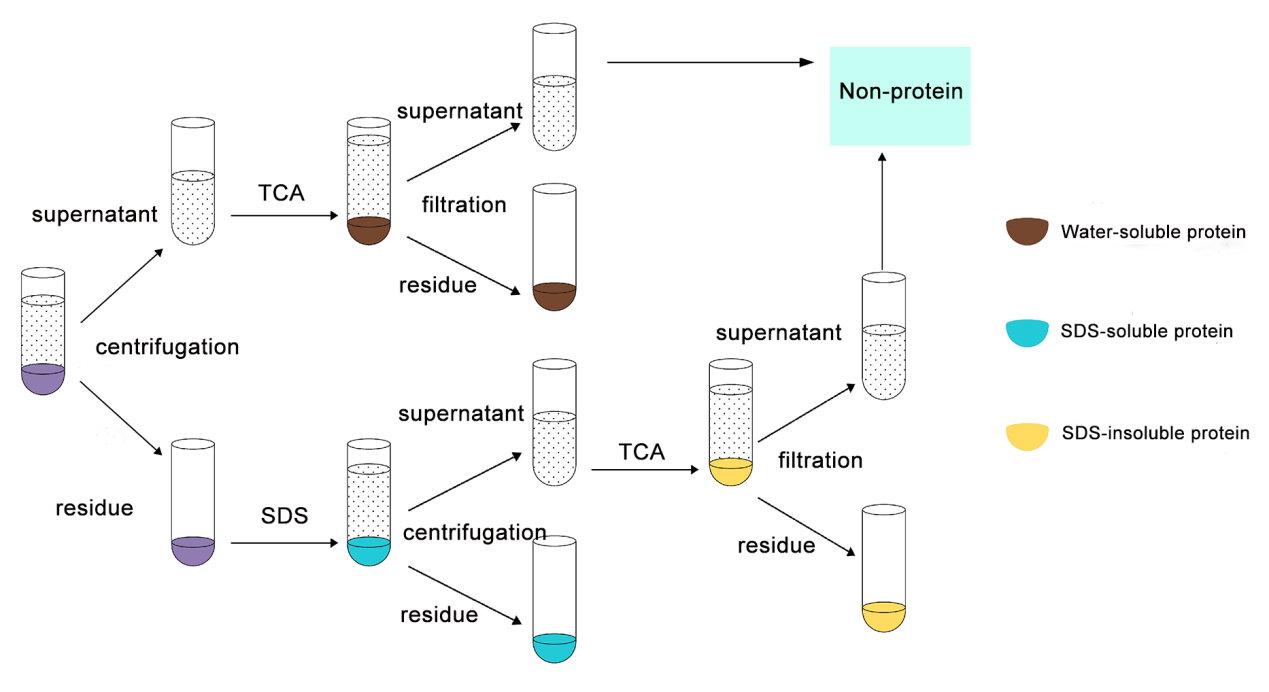


**Figure S2** Flow chart for the extraction of different N morphology. PBS (Na phosphate buffer). TCA (20% trichloroacetic acid), SDS (3% sodium dodecyl sulfate).


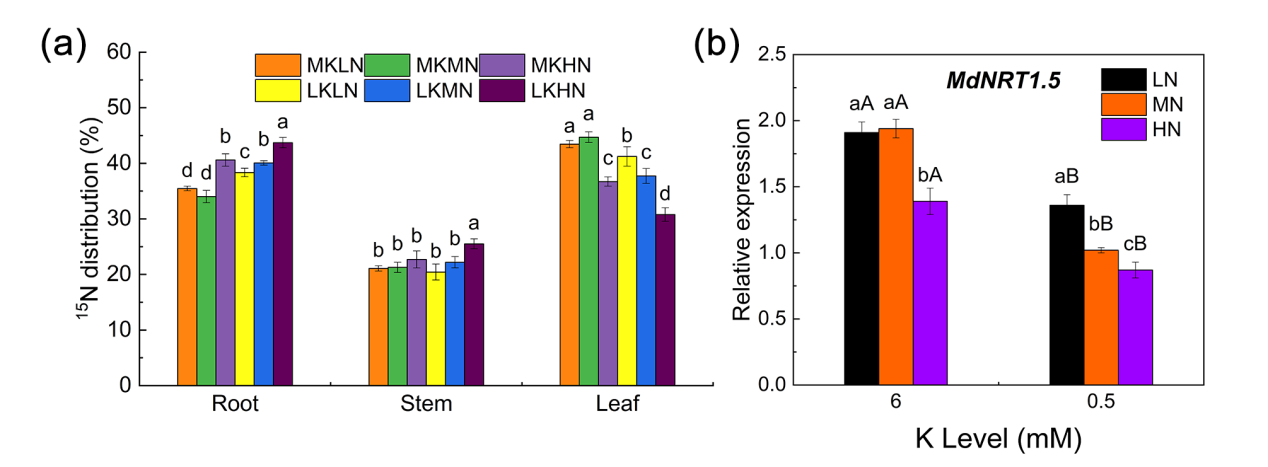


**Figure S3** Effects of different N and K treatment on N distribution of M9T337 rootstock leaves. Different capital letters indicate statistical differences between K treatments under the same N level, whereas different lower case letters indicate statistical differences between N treatments under the same K level (*P* < 0.05).





**Figure S4** Relationship between the net photosynthetic rate (*P*_n_) and relative content of the different N forms.
